# Effect of mechanical surface pretreatment, acid conditioning, and adhesive strategy on the repair microtensile bond strength of hydrolytically aged short fiber-reinforced resin composite: an in vitro study

**DOI:** 10.1186/s12903-026-09956-2

**Published:** 2026-10-12

**Authors:** Mohammed Nasser M. Anwar, Dina Kamal, Heba Mohamed Ahmed Al-Sanafawy

**Affiliations:** 1 Department of Conservative Dentistry, Faculty of Dentistry, Ain Shams University, Cairo, Egypt; 2 Department of Conservative Dentistry, Faculty of Dentistry, Cairo University, Cairo, Egypt; 3 Department of Conservative Dentistry, School of Dentistry, Newgiza University, Giza, Egypt

**Keywords:** Acid conditioning, Failure mode, Hydrolytic aging, Microtensile bond strength, Resin composite repair, Short fiber-reinforced composite, Surface pretreatment, Universal adhesive

## Abstract

**Objectives:**

To evaluate the effect of different mechanical surface pretreatment methods, acid conditioning protocols, and adhesive strategies on the repair microtensile bond strength (µTBS) and failure-mode distribution of hydrolytically aged short fiber-reinforced resin composite (SFRC).

**Materials and methods:**

A total of 144 SFRC specimens were hydrolytically aged in artificial saliva for 3 months and randomly allocated to 12 experimental groups (*n* = 12) according to mechanical surface pretreatment (air abrasion or diamond bur roughening), acid conditioning (37% phosphoric acid or 9.5% hydrofluoric acid), and adhesive strategy (universal adhesive only, silane + universal adhesive, or Acid Resistant Silane [ARS]-containing adhesive). Following the repair procedures, beam-shaped specimens were subjected to µTBS testing and stereomicroscopic failure-mode analysis. Data were analyzed using three-way factorial ANOVA to evaluate the main effects and interactions of mechanical surface pretreatment, acid conditioning, adhesive strategy on µTBS, followed by Tukey’s post-hoc test for pairwise comparisons. Failure-mode data were summarized at the composite-disc level and analyzed using the Monte Carlo permutation chi-square test.

**Results:**

Three-way factorial ANOVA demonstrated significant main effects of acid conditioning and adhesive strategy (both *p* < 0.001), whereas the main effect of mechanical surface pretreatment was not significant (*p* = 0.100). Significant two-way interactions were observed between the treatment factors, whereas the three-way interaction was not significant (*p* = 0.644). The Air abrasion + Phosphoric acid + Universal adhesive protocol demonstrated the highest µTBS value (46.87 ± 4.44 MPa) and predominantly cohesive failures. In contrast, hydrofluoric acid-treated protocols demonstrated significantly lower µTBS values and predominantly adhesive failures. Additional silane application and use of an ARS-containing adhesive did not significantly improve repair bond strength.

**Conclusions:**

A simplified repair protocol combining mechanical surface pretreatment, phosphoric acid conditioning, and universal adhesive application provided favorable repair performance for aged SFRC. Hydrofluoric acid conditioning was associated with lower repair bond strength, while neither additional silanization nor the use of an ARS-containing adhesive provided a significant bond-strength advantage.

**Clinical relevance:**

Simplifying the repair procedure may reduce the number of clinical steps and associated technique sensitivity, potentially facilitating chairside repair of aged SFRC restorations.

## Introduction

Resin-based composite materials are widely used in contemporary restorative dentistry because of their excellent esthetic properties, conservative tooth preparation requirements, and favorable clinical longevity [1]. Despite continuous advancements in monomer chemistry and filler technology, conventional particulate-filled resin composites remain mechanically limited when used for restoration of large stress-bearing posterior cavities [2]. Under high occlusal loading conditions, these materials remain susceptible to polymerization shrinkage stress, microleakage, wear, and insufficient fracture toughness, which may ultimately result in catastrophic bulk fracture or restoration failure [3].

To overcome these structural limitations, short fiber-reinforced resin composites (SFRCs) were introduced as biomimetic dentin-replacement restorative materials for large posterior restorations and structurally compromised teeth [4]. Such materials combine a highly cross-linked semi-interpenetrating polymer network (semi-IPN) matrix with randomly oriented silanated short E-glass fibers and barium silicate particulate fillers. This unique multi-phase structure provides enhanced fracture toughness and crack-stopping ability by allowing the fibers to absorb and redistribute functional stresses while deflecting crack propagation through the restoration [5]. Consequently, SFRCs have demonstrated improved load-bearing capacity and fracture resistance compared with conventional particulate-filled composites, making them particularly suitable for high-stress posterior applications [4, 5].

Despite their superior mechanical performance, resin composite restorations remain susceptible to clinical degradation over time. Hydrolytic aging, thermal fluctuations, occlusal fatigue, marginal deterioration, secondary caries, chipping, and partial structural fractures may necessitate clinical intervention [6]. Traditionally, defective restorations have frequently been managed through complete replacement; however, total restoration replacement is often unnecessarily invasive because it requires removal of sound tooth structure, increases the risk of pulpal irritation or injury, prolongs chairside treatment time, and may contribute to the repetitive restorative cycle that progressively weakens the tooth-restoration complex [7, 8]. Consequently, repair of defective composite restorations has become increasingly advocated as a conservative and minimally invasive alternative to complete replacement [9].

Achieving a durable adhesive repair interface on aged resin composite substrates remains clinically challenging. Over time, prolonged exposure to the oral environment results in hydrolytic degradation, water sorption, resin matrix plasticization, filler-matrix debonding, and deterioration of the superficial resin layer [10]. In addition, polymerization of the original restoration depletes the oxygen-inhibited layer and substantially reduces the number of unreacted carbon double bonds available for direct copolymerization with freshly applied repair composite [11]. Consequently, successful repair bonding becomes increasingly dependent on effective micromechanical surface modification, adequate substrate conditioning, and sufficient adhesive infiltration rather than chemical copolymerization alone [12].

Various mechanical and chemical surface treatment protocols have been investigated to improve bonding to aged resin composites. Mechanical roughening procedures such as airborne-particle abrasion and rotary diamond bur roughening aim to increase surface area and create microretentive irregularities while exposing inorganic filler particles that facilitate micromechanical interlocking [13]. Chemical conditioning procedures, including phosphoric acid and hydrofluoric acid application, have also been investigated to modify surface characteristics and substrate reactivity before adhesive application [14].

Importantly, recent investigations have specifically examined the bonding and repairability of SFRC substrates. Previous research has demonstrated an association between increased surface roughness and improved bonding to SFRC substrates [15], whereas another study reported no significant differences in repaired bond strength among different surface preparation methods and no additional benefit from separate silanization [16]. These findings indicate that SFRC repair is an emerging area of investigation; however, the available evidence remains limited and heterogeneous with respect to the SFRC material, aging protocol, mechanical pretreatment, chemical conditioning, and adhesive strategy evaluated. Moreover, the effects of combining these variables into complete repair workflows have not been comprehensively characterized. This is particularly relevant because the heterogeneous composition of SFRC, comprising a polymer matrix, inorganic fillers, and short glass fibers, may result in a surface response that differs from that of conventional particulate-filled composites and silica-based ceramics. Consequently, the effectiveness of these surface-treatment approaches for repair of aged SFRC substrates remains unclear.

Adhesive strategy may also influence repair performance. Modern universal adhesives, silane-containing systems, and recently introduced Acid Resistant Silane (ARS)-containing adhesives have been proposed to improve adhesion to aged composite substrates [17, 18]. Nevertheless, the effectiveness of these adhesive strategies, particularly when combined with different mechanical and chemical surface treatments, remains insufficiently investigated for hydrolytically aged SFRC substrates.

Important methodological differences remain among existing SFRC repair studies, including the type and duration of aging, mechanical surface pretreatment, chemical conditioning, adhesive formulation, and bond-strength methodology. Furthermore, previous studies have generally focused on selected combinations of surface-treatment variables rather than systematically comparing complete integrated repair protocols incorporating mechanical pretreatment, acid conditioning, and adhesive strategy within the same factorial framework. Such approaches may not fully capture the interaction effects that occur during an actual clinical repair sequence, in which these procedures are performed consecutively. Evaluating complete integrated repair protocols may therefore provide clinically relevant information regarding the combined effects of these procedures and help identify simplified repair strategies for aged SFRC substrates.

Therefore, the aim of the present in vitro study was to systematically evaluate the effect of integrated repair protocols combining mechanical surface pretreatment methods (air abrasion or diamond bur roughening), acid conditioning (37% phosphoric acid or 9.5% hydrofluoric acid), and adhesive strategies (universal adhesive only, silane application followed by universal adhesive, or an Acid Resistant Silane-containing universal adhesive) on the microtensile repair bond strength (µTBS) and failure-mode distribution of hydrolytically aged short fiber-reinforced resin composite. The null hypothesis tested was that variations in the integrated repair protocols would not significantly influence the repair bond strength of aged short fiber-reinforced resin composite restorations.

## Materials and methods

### Study design and sample size calculation

This in vitro study evaluated the effect of different mechanical surface pretreatment methods, acid conditioning protocols, and adhesive strategies on the repair microtensile bond strength (µTBS) of hydrolytically aged short fiber-reinforced resin composite.

Sample size was calculated using G*Power software (version 3.1.9.7; Heinrich-Heine University, Düsseldorf, Germany) for a fixed-effects factorial ANOVA with a 2 × 2 × 3 factorial design, representing 12 experimental groups and the effects of mechanical surface pretreatment, acid conditioning, and adhesive strategy on µTBS. An F-test [ANOVA: fixed effects, special, main effects and interactions] was selected with an anticipated large effect size (f = 0.40), a significance level of α = 0.05, and statistical power of 80% (1 − β = 0.80), with 11 numerator degrees of freedom. The calculation yielded a minimum required sample size of 116 experimental units. A 25% allowance was subsequently applied to account for potential specimen loss or reduced statistical efficiency, resulting in a target sample size of 145 experimental units. The sample was rounded for equal allocation across the 12 groups, and 144 composite discs (12 discs per group) were ultimately included. Each composite disc was considered one experimental unit for the primary µTBS analysis, while multiple beams obtained from each disc were treated as subsamples. The composite disc was also considered the independent experimental unit for failure-mode analysis, with beam-level failure-mode assessments treated as subsamples nested within the corresponding disc.

### Materials used

The proprietary designations, descriptions, manufacturers, chemical compositions, and lot numbers of the materials investigated in this study are compiled in Table 1.

**Table 1:** Materials used in the study and their manufacturers, compositions, and descriptions

Trade Name	Material Description	Manufacturer	Composition	Lot Number
everX Posterior	Short fiber-reinforced composite	GC Corporation, Tokyo, Japan	Bis-GMA, TEGDMA, PMMA, Silanated E-glass fibers (17 μm diameter, 1–2 mm length, 9 wt%), Barium silicate glass fillers (67 wt%), Fumed silicon dioxide (SiO_2_), Camphorquinone, Tertiary amine, Diphenyl (2,4,6-trimethylbenzoyl) phosphine oxide (TPO)	2508011
All-Bond Universal	One-bottle universal dental adhesive system	Bisco Inc., Schaumburg IL, USA	10-MDP, Bis-GMA, HEMA, Ethanol/water solvent system (~ 2.5 wt% water), Camphorquinone, Tertiary amine (ethyl-4-dimethylaminobenzoate system). Ultra-mild acidity (pH = 3.2)	2500007558
Porcelain Primer	Single-component pre-hydrolyzed silane coupling agent	Bisco Inc., Schaumburg IL, USA	3-(Trimethoxysilyl) propyl-2-methyl-2-propenoic acid (methacrylate functional silane monomer), Acetone/ethanol anhydrous solvent system, Acetic acid catalyst	2500005483
BeautiBond Xtreme	One-step, filler-free, self-etch ARS-containing universal adhesive	Shofu Dental Corporation, Kyoto, Japan	Phosphonated/Carboxylated monomers, Quadra functional monomer system with ARS coupling agents, Bis-GMA, TEGDMA, Acetone/water solvent system, Camphorquinone-based photoinitiators	122234
OptiBond Gel Etchant	Enamel/dentin conditioning gel	Kerr Corporation, Orange, CA, USA	37 wt% Phosphoric acid (H_3_PO_4_), Water, Polymeric thickener/gelling agent, Blue pigment coloring agent	12886644
Porcelain Etchant	Ceramic micro-mechanical surface etchant gel	Bisco Inc., Schaumburg IL, USA	9.5 wt% Hydrofluoric acid (HF), Water, Buffering salts, polymeric gelling agent, Thickener	2500005648
AquaCare Air Abrasion System	Air abrasion device	Velopex International, London, UK	Synthetic alpha-aluminum oxide abrasive powder particle stream (α-Al_2_O_3_, 29-µm nominal grit sizing)	—

*Bis-GMA* bisphenol A-glycidyl methacrylate, *TEGDMA* triethylene glycol dimethacrylate, *PMMA* polymethyl methacrylate, *MDP* 10-methacryloyloxydecyl dihydrogen phosphate, *HEMA* hydroxyethyl methacrylate, *ARS* Acid Resistant Silane

### Specimen fabrication

A total of 144 disc-shaped specimens were fabricated from a short fiber-reinforced resin composite (everX Posterior, GC Corporation, Tokyo, Japan). Custom-made, split stainless-steel molds with standardized cylindrical cavities (8 mm in diameter and 4 mm in height) were positioned on a polyethylene-insulated glass slab and used for specimen fabrication. The composite material was packed into the molds in two successive 2-mm increments. Each increment was photo-polymerized for 20 s using an LED light-curing unit (Elipar S10, 3M Oral Care, St. Paul, MN, USA) operating at an irradiance of 1200 mW/cm^2^. The irradiance was periodically verified using a digital radiometer (Hilux Dental Radiometer, Benlioglu Dental, Ankara, Turkey). The curing tip was held perpendicular to the specimen surface in close proximity without direct contact.

The final composite increment was covered with a transparent Mylar strip and compressed with a glass slide to obtain a flat surface and reduce formation of the oxygen-inhibited layer. After removal from the molds, additional light-curing was performed from the top and bottom surfaces for 20 s each to ensure adequate polymerization.

The bonding surfaces were subsequently finished and polished using an aluminum oxide-impregnated silicone polishing system (OneGloss, Shofu Dental Corporation, Kyoto, Japan) under water cooling with a low-speed handpiece for 20 s per specimen. All specimens were prepared by a single calibrated operator to ensure standardization.

### Artificial aging procedure

To simulate hydrolytic aging, all fabricated composite specimens were immersed in artificial saliva containing 1.5 mM CaCl₂, 0.9 mM NaH₂PO₄, and 0.15 M KCl at pH = 7.0 [19]. The specimens were stored in sealed containers at 37 ℃ for 3 months. The storage medium was renewed daily throughout the aging period [20].

### Experimental grouping design

**Fig. 1:**
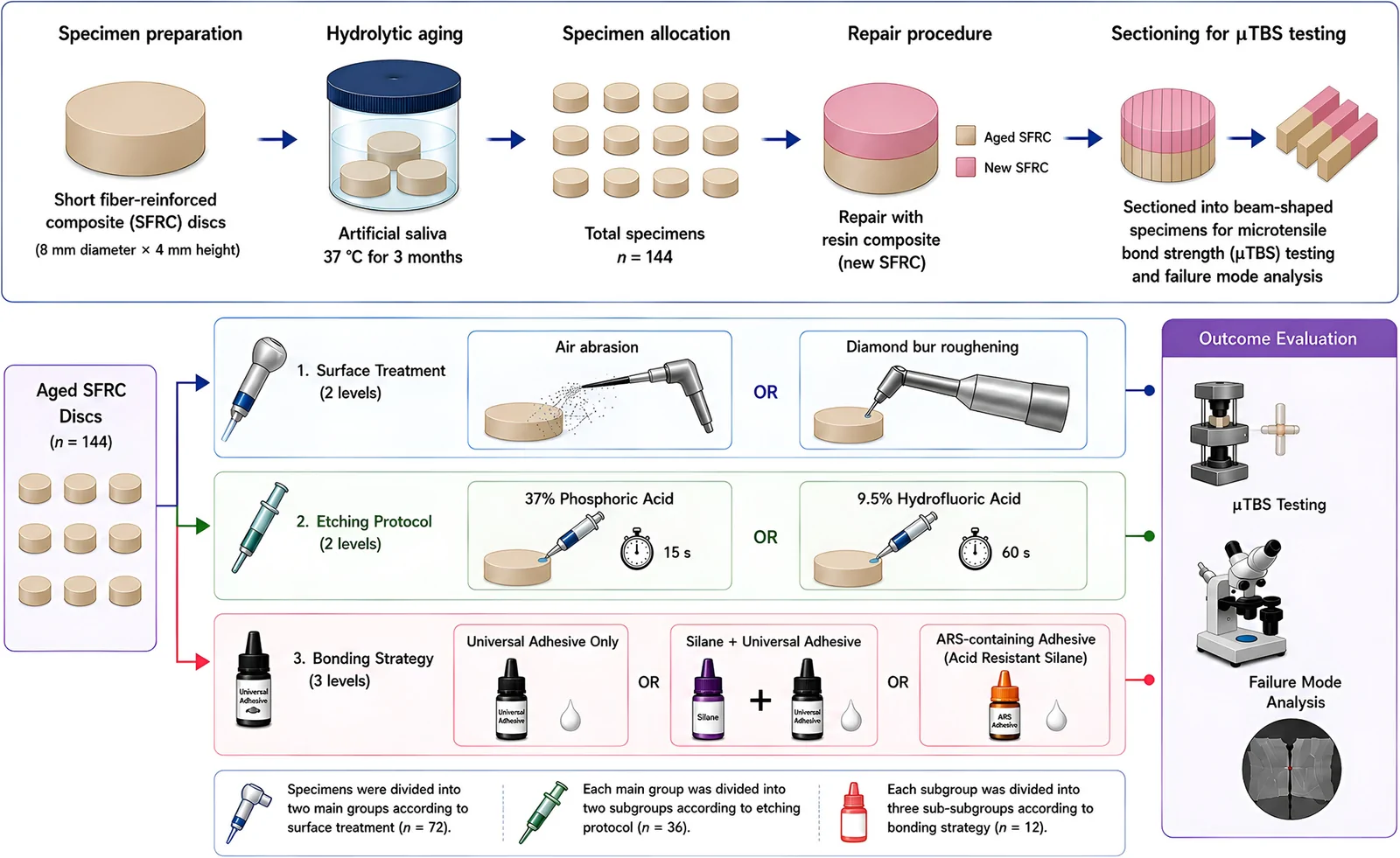
Schematic illustration of the experimental grouping design and workflow

Following artificial aging, the specimens were randomly assigned to two main groups (*n* = 72) according to the mechanical surface pretreatment method: air abrasion or diamond bur roughening. Each main group was further subdivided into two subgroups according to the acid conditioning protocol: 37% phosphoric acid or 9.5% hydrofluoric acid (*n* = 36). Subsequently, each subgroup was further divided into three sub-subgroups based on the bonding strategy: universal adhesive, silane application followed by universal adhesive, or ARS-containing universal adhesive (*n* = 12). This resulted in a total of 12 experimental groups. This experimental grouping design and procedural workflow are illustrated in Fig. 1.

### Mechanical surface pretreatment

#### Air abrasion protocol

Specimens assigned to the air abrasion group (*n* = 72) were treated using an intraoral air abrasion device (AquaCare, Velopex International, London, UK) with 29-µm aluminum oxide particles at a pressure of 2.5 bar. The nozzle was positioned perpendicular to the composite surface at 10 mm distance for 10 s using a continuous, sweeping circular motion. The treated surfaces were then rinsed with air-water spray for 30 s.

#### Diamond Bur roughening protocol

Specimens assigned to the diamond bur group (*n* = 72) were roughened using medium-grit, blue-coded cylindrical diamond burs (TR-25 Dia-Burs, Mani Inc., Tochigi, Japan) in a high-speed handpiece under continuous water cooling. Surface roughening was standardized by delivering exactly 10 unidirectional linear strokes across the face of the specimen by a single calibrated operator. To minimize the confounding effect of structural bur wear, a new diamond bur was substituted after every fifth specimen preparation.

### Post-pretreatment ultrasonic cleaning

Immediately following their respective mechanical treatments, all 144 specimens across both groups were completely submerged in an ultrasonic cleaning bath filled with fresh distilled water. The specimens were cleaned continuously for 5 min to remove residual surface contaminants generated during mechanical pretreatment. Following ultrasonic cycling, all treated surfaces were thoroughly dried using a strong stream of oil-free compressed air for 10 s prior to the subsequent acid conditioning and adhesive protocols.

### Acid conditioning and adhesive strategies

#### Acid conditioning protocols

For phosphoric acid-treated groups, the mechanically pretreated composite surfaces were etched with 37% phosphoric acid gel (OptiBond Gel Etchant, Kerr Corporation, CA, USA) for 15 s, rinsed thoroughly with water for 20 s, and air dried for 10 s. For hydrofluoric acid-treated groups, the pretreated composite surfaces were etched with hydrofluoric acid gel (Porcelain Etchant, Bisco Inc., IL, USA) for 60 s, rinsed with water for 60 s, and air dried for 10 s.

#### Adhesive strategies

- Universal Adhesive-Only Strategy: Two consecutive coats of the universal adhesive (All-Bond Universal, Bisco Inc., IL, USA) were applied and actively rubbed for 10–15 s per coat without intermediate light-curing. Excess solvent was evaporated using a stream of oil-free compressed air for 10 s until a uniform glossy surface was obtained. The adhesive layer was then photo-polymerized for 10 seconds.
- Silane + Universal Adhesive Strategy: A silane coupling agent (Porcelain Primer, Bisco Inc., IL, USA) was applied to the etched surfaces, allowed to react undisturbed for 30 s, and then gently air dried. Subsequently, the universal adhesive was applied according to the same protocol described for the adhesive-only strategy.
- ARS-containing Universal Adhesive Strategy: The ARS-containing universal adhesive (BeautiBond Xtreme, Shofu Dental Corporation, Kyoto, Japan) was applied to the treated surfaces and actively rubbed for 20 s. Gentle air was applied for 3 s followed by stronger air drying until a uniform glossy surface with no visible adhesive movement was obtained. The adhesive layer was then light cured for 10 s.

### Repair procedure and resin composite placement

Following adhesive application, the repair composite was applied over the aged composite substrates using the same short fiber-reinforced resin composite material. The repair composite was placed in two successive 2-mm increments to obtain a final height of 4 mm. Each increment was photo-polymerized for 20 s using the LED light-curing unit at an irradiance of 1200 mW/cm^2^. The repaired composite discs were then stored in distilled water at 37 ℃ for 24 h before sectioning and testing.

### Microtensile bond strength (µTBS) testing

After 24 h of water storage, the repaired composite discs were embedded in auto-polymerizing acrylic resin and secured within a high-precision gripping fixture to guarantee rigid, standardized axial alignment during sectioning procedures. Serial longitudinal sectioning was executed using a low-speed automated diamond saw (Isomet 4000, Buehler Ltd., Lake Bluff, IL, USA) under copious water cooling supplemented with a water-soluble, anticorrosive lubricant (Cool 2, Buehler Ltd., Lake Bluff, IL, USA) diluted at a 1:33 lubricant-to-water volume ratio.

Serial sectioning was first performed in one direction, followed by a second perpendicular sectioning after rotating the specimen assembly by 90°. Sectioning was performed using a 0.3-mm-thick diamond-coated wafering blade to obtain beam-shaped specimens with an approximate cross-sectional area of 1 mm × 1 mm at the bonded interface. Beam dimensions were verified using a digital caliper (TOTAL Tools, Selangor, Malaysia). Following serial sectioning, each repaired composite disc yielded approximately 24–28 beam-shaped specimens. Peripheral beams exhibiting irregular geometry or sectioning defects were excluded before testing, resulting in approximately 4–8 excluded beams and 20 valid beams per composite disc. No pre-test failures of valid beams occurred during specimen handling or testing. The mean µTBS of all valid beams obtained from each repaired composite disc was calculated and considered one experimental unit for statistical analysis, thereby accounting for the clustering of beams within each composite disc. Thus, individual beams were treated as subsamples nested within their respective composite discs rather than as independent experimental units.

Each beam was attached to a microtensile testing jig using cyanoacrylate adhesive (Akfix 705 Fast Adhesive, Istanbul, Turkey), ensuring that the adhesive remained at least 1 mm away from the bonded interface. The jig was mounted in a universal testing machine (Instron Model 3345, Instron Corp., Canton, MA, USA) equipped with a 500-N load cell. Tensile load was applied at a crosshead speed of 0.5 mm/min until failure occurred [21].

The microtensile bond strength (µTBS) was calculated in megapascals (MPa) using the following equation:

$$\upmu\mathrm{TBS}=\mathrm{F/A}$$

where F represents the maximum load at failure (N) and A represents the bonded cross-sectional area (mm²). 

### Failure mode analysis

Following µTBS testing, the fractured surfaces of all beams were examined under a stereomicroscope (XCAM, 1080P8MPB Stereomicroscope, ToupTek Photonics, Zhejiang, China) at ×30 magnification. Failure modes were classified by a single examiner as:

- Adhesive failure: characterized by complete separation along the interface between the aged and repair composites without fracture of either substrate.
- Cohesive failure: characterized by fracture occurring entirely within either the aged composite substrate or the repair composite.
- Mixed failure: characterized by partial adhesive debonding along the interface accompanied by localized cohesive fracture within either the aged composite substrate or the repair composite.

Because multiple beams were obtained from each composite disc, beam-level failure-mode observations originating from the same disc were considered subsamples and were not treated as statistically independent observations for inferential analysis. For each composite disc, a single disc-level failure-mode classification was assigned based on the failure-mode category occurring most frequently among its classified beams. The resulting disc-level classifications were used as the independent observations for statistical analysis, with the 12 composite discs representing the independent experimental units within each repair protocol.

To ensure consistent application of the predefined failure-mode criteria, the examiner underwent calibration prior to the assessment using 20 representative specimens. Furthermore, failure-mode assessment was performed under blinded conditions, with the experimental group allocation concealed using randomized alphanumeric labels assigned by a third party.

### Statistical analysis

Statistical analysis was performed using MedCalc Statistical Software (version 22; MedCalc Software Ltd., Ostend, Belgium). Normality of each group was assessed using the Shapiro–Wilk test. All 12 groups showed a normal distribution (*p* > 0.05). µTBS data were expressed as mean ± standard deviation (SD). The mean µTBS of all valid beams obtained from each composite disc was calculated, and the composite disc was treated as the experimental unit. A three-way factorial ANOVA was performed to evaluate the main effects of mechanical surface pretreatment (air abrasion versus diamond bur), acid conditioning (phosphoric acid versus hydrofluoric acid), and adhesive strategy (universal adhesive, silane + universal adhesive, and ARS-containing universal adhesive), as well as all two-way and three-way interaction effects. Tukey’s post-hoc test was used for pairwise comparisons among the 12 experimental groups. Because beams obtained from the same disc were subsamples and could not be considered statistically independent, failure-mode data were summarized at the composite-disc level, with each disc contributing one categorical observation based on the failure-mode category occurring most frequently among its classified beams. Disc-level failure-mode distributions among the 12 repair protocols were compared using the Monte Carlo permutation chi-square test because several cells contained low or zero frequencies. Statistical significance was set at *p* < 0.05.

## Results

### Microtensile bond strength (µTBS)

Three-way factorial ANOVA demonstrated a highly significant main effect of acid conditioning (F = 1237.44, *p* < 0.001) and adhesive strategy (F = 16.39, *p* < 0.001), whereas the main effect of mechanical surface pretreatment was not statistically significant (F = 2.75, *p* = 0.100) (Table 2). Significant two-way interactions were observed between mechanical surface pretreatment and acid conditioning (F = 5.87, *p* = 0.017), mechanical surface pretreatment and adhesive strategy (F = 8.60, *p* < 0.001), and acid conditioning and adhesive strategy (F = 18.16, *p* < 0.001). However, the three-way interaction among mechanical surface pretreatment, acid conditioning, and adhesive strategy was not statistically significant (F = 0.44, *p* = 0.644).

**Table 2:** Three-way factorial ANOVA showing the main and interaction effects of mechanical surface pretreatment, acid conditioning, and adhesive strategy on µTBS

Effect	F	*p*-value
Mechanical surface pretreatment	2.75	0.100
Acid conditioning	1237.44	< 0.001^*^
Adhesive strategy	16.39	< 0.001^*^
Mechanical surface pretreatment × Acid conditioning	5.87	0.017^*^
Mechanical surface pretreatment × Adhesive strategy	8.60	< 0.001^*^
Acid conditioning × Adhesive strategy	18.16	< 0.001^*^
Mechanical surface pretreatment × Acid conditioning × Adhesive strategy	0.44	0.644

^*^Statistically significant at *p*  < 0.05

The mean µTBS values and standard deviations (SD) for the 12 evaluated repair protocols are presented in Table 3. Tukey’s post-hoc comparisons among the 12 repair protocols showed that the Air Abrasion + Phosphoric Acid + Universal Adhesive protocol demonstrated the highest µTBS (46.87 ± 4.44 MPa) and was significantly higher than all other evaluated protocols (*p* < 0.05). The Diamond Bur + Phosphoric Acid + Universal Adhesive protocol had the second-highest mean µTBS (39.84 ± 4.00 MPa).

**Table 3:** Mean ± standard deviation (SD) of µTBS values (MPa) for the evaluated repair protocols

Repair Protocol	Mean ± SD (MPa)
Air abrasion + Phosphoric acid + Universal adhesive	46.87 ± 4.44^a^
Diamond bur + Phosphoric acid + Universal adhesive	39.84 ± 4.00^b^
Diamond bur + Phosphoric acid + Silane + Universal adhesive	37.77 ± 4.10^bc^
Air abrasion + Phosphoric acid + Silane + Universal adhesive	37.33 ± 4.47^bc^
Air abrasion + Phosphoric acid + ARS-containing adhesive	34.75 ± 4.37^bc^
Diamond bur + Phosphoric acid + ARS-containing adhesive	33.17 ± 3.09^c^
Diamond bur + Hydrofluoric acid + Silane + Universal adhesive	16.62 ± 4.06^d^
Air abrasion + Hydrofluoric acid + Universal adhesive	15.92 ± 4.79^d^
Diamond bur + Hydrofluoric acid + ARS-containing adhesive	15.79 ± 4.19^d^
Air abrasion + Hydrofluoric acid + Silane + Universal adhesive	14.73 ± 3.98^d^
Air abrasion + Hydrofluoric acid + ARS-containing adhesive	13.20 ± 3.57^d^
Diamond bur + Hydrofluoric acid + Universal adhesive	12.96 ± 2.17^d^

Means sharing at least one common lowercase superscript letter were not significantly different according to Tukey’s post-hoc test (*p* ≥ 0.05)

Among the remaining phosphoric acid-treated protocols, the Diamond Bur + Phosphoric Acid + Silane + Universal Adhesive (37.77 ± 4.10 MPa), Air Abrasion + Phosphoric Acid + Silane + Universal Adhesive (37.33 ± 4.47 MPa), and Air Abrasion + Phosphoric Acid + ARS-containing Adhesive (34.75 ± 4.37 MPa) protocols showed comparable mean µTBS values. The Diamond Bur + Phosphoric Acid + ARS-containing Adhesive protocol had the lowest numerical mean µTBS among the phosphoric acid-treated protocols (33.17 ± 3.09 MPa).

All hydrofluoric acid-treated protocols demonstrated significantly lower µTBS values than the phosphoric acid-treated protocols. Mean µTBS values among the hydrofluoric acid-treated protocols ranged from 12.96 ± 2.17 MPa for the Diamond Bur + Hydrofluoric Acid + Universal Adhesive protocol to 16.62 ± 4.06 MPa for the Diamond Bur + Hydrofluoric Acid + Silane + Universal Adhesive protocol. No significant differences were detected among the hydrofluoric acid-treated protocols (*p* ≥ 0.05). 

### Failure mode analysis

The disc-level frequency distributions of adhesive, mixed, and cohesive failure modes among the 12 repair protocols are presented in Table 4. The distribution of failure modes differed significantly among the repair protocols according to the Monte Carlo permutation chi-square test (*p* < 0.001).

**Table 4:** Disc-level distribution of failure modes for the evaluated repair protocols, *n* (%)

Repair protocol	Adhesive	Mixed	Cohesive
Air abrasion + Phosphoric acid + Universal adhesive	1 (8.3%)	2 (16.7%)	9 (75.0%)
Air abrasion + Phosphoric acid + Silane + Universal adhesive	3 (25.0%)	2 (16.7%)	7 (58.3%)
Air abrasion + Phosphoric acid + ARS-containing adhesive	2 (16.7%)	3 (25.0%)	7 (58.3%)
Diamond bur + Phosphoric acid + Universal adhesive	3 (25.0%)	6 (50.0%)	3 (25.0%)
Diamond bur + Phosphoric acid + Silane + Universal adhesive	3 (25.0%)	7 (58.3%)	2 (16.7%)
Diamond bur + Phosphoric acid + ARS-containing adhesive	5 (41.7%)	4 (33.3%)	3 (25.0%)
Air abrasion + Hydrofluoric acid + Universal adhesive	10 (83.3%)	2 (16.7%)	0 (0.0%)
Air abrasion + Hydrofluoric acid + Silane + Universal adhesive	12 (100.0%)	0 (0.0%)	0 (0.0%)
Air abrasion + Hydrofluoric acid + ARS-containing adhesive	12 (100.0%)	0 (0.0%)	0 (0.0%)
Diamond bur + Hydrofluoric acid + Universal adhesive	12 (100.0%)	0 (0.0%)	0 (0.0%)
Diamond bur + Hydrofluoric acid + Silane + Universal adhesive	11 (91.7%)	1 (8.3%)	0 (0.0%)
Diamond bur + Hydrofluoric acid + ARS-containing adhesive	10 (83.3%)	2 (16.7%)	0 (0.0%)
*p*-value	< 0.001*		

^*^Statistically significant at *p* < 0.05

Among the phosphoric acid-treated protocols, cohesive failure predominated in the Air Abrasion + Phosphoric Acid + Universal Adhesive protocol, occurring in 9 of 12 discs (75.0%). Cohesive failure also predominated in the Air Abrasion + Phosphoric Acid + Silane + Universal Adhesive and Air Abrasion + Phosphoric Acid + ARS-containing Adhesive protocols, occurring in 7 of 12 discs (58.3%) in each protocol. In contrast, mixed failure predominated in the Diamond Bur + Phosphoric Acid + Universal Adhesive and Diamond Bur + Phosphoric Acid + Silane + Universal Adhesive protocols, occurring in 6 of 12 discs (50.0%) and 7 of 12 discs (58.3%), respectively. The Diamond Bur + Phosphoric Acid + ARS-containing Adhesive protocol showed a more balanced distribution of adhesive, mixed, and cohesive failures, occurring in 5 (41.7%), 4 (33.3%), and 3 (25.0%) discs, respectively.

**Fig. 2:**
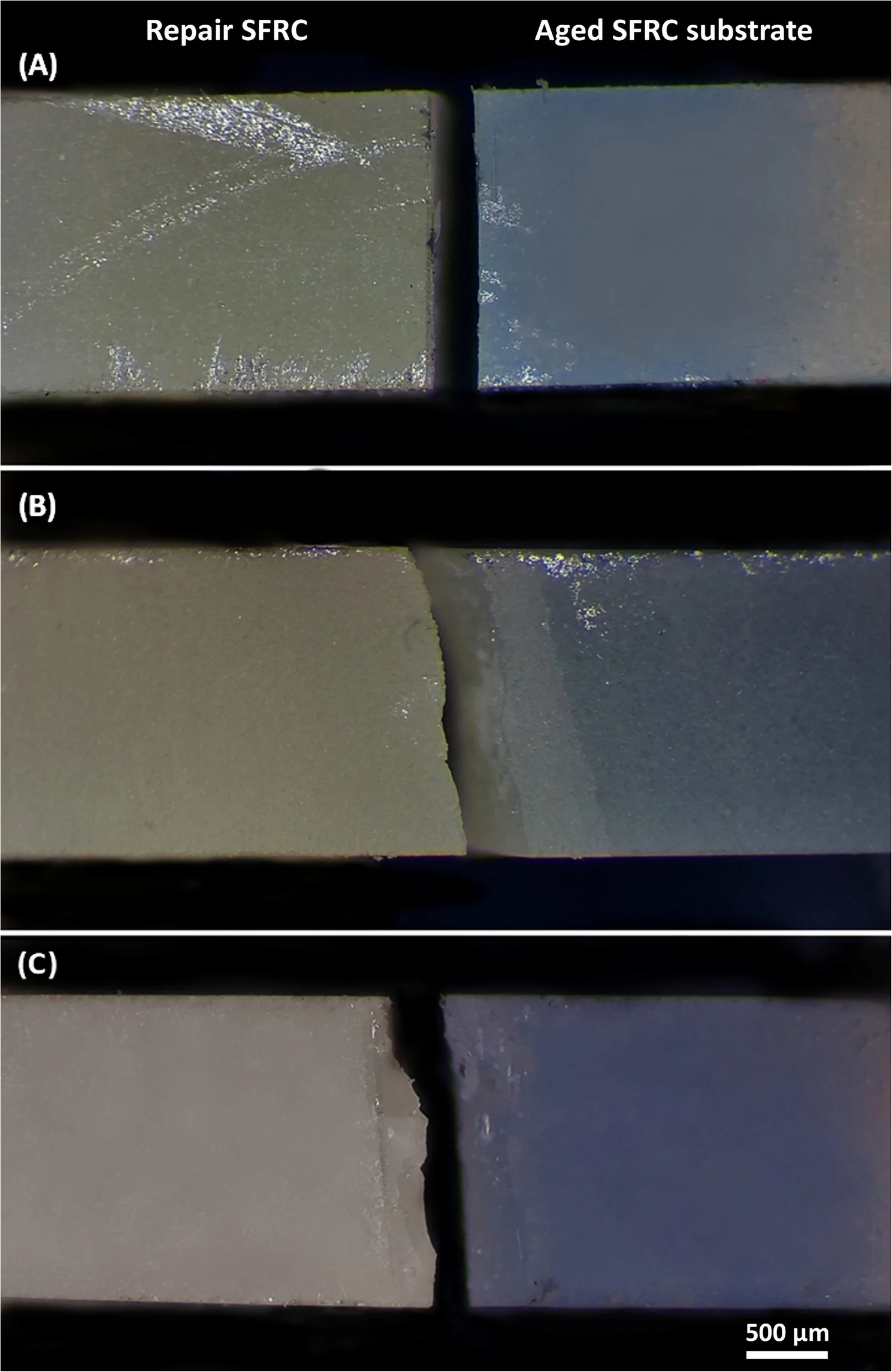
Representative stereomicroscopic images (×30 magnification) illustrating adhesive (**A**), cohesive (**B**), and mixed (**C**) failure modes following microtensile bond strength testing. “Repair SFRC” and “Aged SFRC Substrate” indicate the respective sides of the fractured repair specimens

Hydrofluoric acid-treated protocols demonstrated a marked predominance of adhesive failure. All 12 discs (100.0%) showed adhesive failure in the Air Abrasion + Hydrofluoric Acid + Silane + Universal Adhesive, Air Abrasion + Hydrofluoric Acid + ARS-containing Adhesive, and Diamond Bur + Hydrofluoric Acid + Universal Adhesive protocols. The Air Abrasion + Hydrofluoric Acid + Universal Adhesive and Diamond Bur + Hydrofluoric Acid + ARS-containing Adhesive protocols each showed adhesive failure in 10 of 12 discs (83.3%), while the remaining 2 discs (16.7%) showed mixed failure. The Diamond Bur + Hydrofluoric Acid + Silane + Universal Adhesive protocol showed adhesive failure in 11 of 12 discs (91.7%) and mixed failure in 1 disc (8.3%). Representative stereomicroscopic images of the observed failure modes are presented in Fig. 2. 

## Discussion

The present in vitro study evaluated the effects of mechanical surface pretreatment, acid conditioning, and adhesive strategy on the repair microtensile bond strength (µTBS) of hydrolytically aged short fiber-reinforced resin composite (SFRC). By evaluating complete repair protocols within a three-factorial experimental design, the study aimed to identify clinically applicable and simplified approaches for repair of aged SFRC substrates. The three-way factorial analysis demonstrated significant main effects of acid conditioning and adhesive strategy, whereas the overall main effect of mechanical surface pretreatment was not significant. Significant two-way interactions were observed, indicating that the effect of one treatment factor varied according to the level of another factor. The absence of a significant three-way interaction suggests that no additional interaction involving all three factors simultaneously was detected. Based on the significant differences among the evaluated repair protocols, the null hypothesis was rejected.

Although SFRCs are primarily indicated as dentin-replacement/core materials and are commonly covered with conventional particulate-filled composite, the present study specifically investigated the repairability of the SFRC substrate itself. This material-specific approach was intended to characterize the bonding response of aged SFRC to different repair protocols rather than imply that SFRC is routinely used as the final exposed restorative material. The heterogeneous structure of SFRC, comprising a polymer matrix, inorganic fillers, and short glass fibers, provides a distinct substrate for repair bonding and therefore warrants specific evaluation.

Successful repair of aged resin composites remains clinically challenging because aging results in depletion of the oxygen-inhibited layer and reduction of unreacted carbon double bonds available for direct copolymerization with freshly applied composite. In addition, prolonged hydrolytic degradation promotes water sorption, resin plasticization, filler–matrix debonding, and deterioration of the superficial resin matrix [18]. Consequently, repair bonding becomes increasingly dependent on effective micromechanical surface modification, substrate conditioning, and adhesive infiltration rather than chemical copolymerization alone [17, 18].

Among the evaluated repair protocols, air abrasion followed by phosphoric acid conditioning and application of universal adhesive demonstrated the highest µTBS value. However, the factorial analysis showed that mechanical surface pretreatment did not exert a significant overall main effect, whereas acid conditioning and adhesive strategy were significant factors. The significant two-way interactions further indicate that the effect of mechanical pretreatment varied according to the accompanying acid conditioning and adhesive strategy. In contrast, the absence of a significant three-way interaction indicates that no additional interaction involving all three factors simultaneously was detected. Thus, the superior performance of the air abrasion + phosphoric acid + universal adhesive protocol should be interpreted as the outcome of this specific combination of treatment factors rather than as evidence that air abrasion alone is superior to diamond bur roughening.

The superior performance of the air abrasion + phosphoric acid + universal adhesive protocol may reflect the combined effects of mechanical surface modification, acid conditioning, and adhesive application. Mechanical surface pretreatment, whether by air abrasion or rotary diamond bur roughening, may remove the superficial degraded resin layer while increasing surface irregularities and the available bonding area [22–24]. In SFRC substrates, these procedures may also expose underlying inorganic components, including short E-glass fibers and barium silicate fillers, potentially creating surface features favorable for adhesive penetration and micromechanical interlocking [25]. Previous studies have reported beneficial effects of both approaches on repair bonding of aged resin composites, including SFRCs [16, 24–28].

Subsequent phosphoric acid application may have contributed to the favorable bonding performance through surface conditioning rather than an aggressive etching effect. Previous studies have proposed potential benefits of phosphoric acid conditioning following mechanical roughening, including the removal of residual debris, increased surface energy, and improved substrate wettability, which may facilitate adhesive adaptation [13, 15, 28]. Although the present study included a standardized 5-min ultrasonic cleaning step before acid application, the superior performance of these phosphoric acid-treated protocols may reflect the cumulative benefits of surface conditioning and its potential to improve substrate reactivity within the sequential repair workflow. Unlike hydrofluoric acid, phosphoric acid does not aggressively dissolve the structural inorganic phase or disrupt the resin–filler interface, which may have contributed to the higher repair bond strength observed with phosphoric acid-treated protocols [28, 29]. Finally, the favorable performance of the universal adhesive may be related to its functional monomer chemistry, specifically 10-MDP, which has been reported to promote chemical interaction with the inorganic components of resin-based substrates and contribute to interfacial bonding [30].

A notable finding was that separate silane application did not significantly improve repair bond strength compared with the universal adhesive-only protocol. Silane coupling agents are traditionally used to promote bonding to silica-rich inorganic surfaces through siloxane formation [30, 31]. However, aged SFRC presents a heterogeneous substrate comprising a polymer matrix, inorganic fillers, and short glass fibers, with its surface chemistry potentially altered by hydrolytic aging.

Hydrolytic degradation may affect silane-mediated interactions at the fiber–matrix and filler–matrix interfaces, thereby altering the availability or reactivity of surface sites required for stable siloxane formation [32, 33]. Another possible explanation is that application of a separate silane layer may increase interfacial thickness. Thicker interfacial layers may be more susceptible to failure because the repair interface represents the weakest region of repaired composite restorations [17, 18]. In addition, contemporary universal adhesives may contain functional monomers capable of interacting with exposed inorganic components, potentially reducing the incremental benefit of a separate silanization step [34]. Consistent with previous reports [16–18, 34, 35], the present findings therefore suggest that separate silanization may not provide an additional bond-strength advantage when an appropriate surface pretreatment and universal adhesive are used.

The ARS-containing universal adhesive did not demonstrate superior repair performance compared with the conventional universal adhesive used in the present study. However, this finding should be interpreted as a comparison between two distinct commercial adhesive formulations rather than as an evaluation of the isolated effect of ARS technology. The formulations differ in their monomer composition, functional monomers, solvents, and other components in addition to the presence of ARS. Therefore, the present design cannot determine whether the observed difference was specifically attributable to ARS technology.

Although ARS technology is intended to improve silane stability within acidic one-bottle adhesive formulations, several factors may explain the limited benefit. Within simplified adhesives, acidic monomers and silane components coexist, and the acidic environment may influence silane stability and limit effective silane condensation, thereby potentially affecting silane-mediated coupling at the bonding interface [36–38]. These mechanisms remain speculative in the absence of direct chemical or interfacial characterization. Thus, the present findings indicate only that the ARS-containing adhesive did not provide a measurable bond-strength advantage over the conventional universal adhesive under the conditions tested.

A major finding of the present study was the substantially lower performance of all hydrofluoric acid-treated repair protocols compared with the phosphoric acid-treated protocols. Hydrofluoric acid is effective for conditioning silica-based glass ceramics because it selectively dissolves the glassy phase and creates micromechanical porosities favorable for bonding [27, 39]. However, SFRC is a heterogeneous resin-based material containing an organic polymer matrix, inorganic fillers, and short E-glass fibers, and therefore may not respond to hydrofluoric acid in the same manner as glass ceramics.

Previous studies have reported that hydrofluoric acid treatment may adversely affect resin composite repair by eroding exposed filler particles and disrupting the filler–matrix interface. Such alterations may increase surface defects and expose a larger resin-rich area susceptible to hydrolytic degradation. In fiber-reinforced composite systems, hydrofluoric acid conditioning has likewise been reported to provide no improvement in repair bond strength and may be detrimental when excessive dissolution of inorganic components comprises the surface structure [17, 27, 40–42]. Consistent with these findings, all hydrofluoric acid-treated protocols in the present study demonstrated substantially lower µTBS values and predominantly adhesive failure patterns. Thus, hydrofluoric acid conditioning did not provide a bonding advantage over phosphoric acid conditioning for aged SFRC.

The failure-mode analysis provided additional context for interpretation of the µTBS findings. Protocols associated with higher bond strength values predominantly exhibited cohesive or mixed failures, whereas hydrofluoric acid-treated protocols showed mainly adhesive failures. Cohesive failures indicate that the repair interface was at least as strong as, or stronger than, the surrounding composite substrate and are therefore consistent with a relatively strong repair interface. However, they also indicate that failure occurred within the substrate rather than at the interface. Consequently, µTBS values from specimens exhibiting cohesive failure should be interpreted as substrate-limited estimates rather than direct measures of ultimate interfacial strength. This consideration is particularly relevant to the highest-strength protocol, in which cohesive failure accounted for 75% of discs.

The findings of the present study are generally consistent with previous investigations of SFRC bonding and repair [15, 16, 27, 43]. A previous SFRC repair study reported no significant differences among different mechanical surface-preparation methods and no additional benefit from separate silanization [16]. A recent investigation of an SFRC CAD/CAM material reported higher shear bond strength following airborne-particle abrasion alone compared with airborne-particle abrasion followed by hydrofluoric acid conditioning [43]. This finding supports the present observation that hydrofluoric acid conditioning did not provide a bonding advantage for aged SFRC repair. These findings are also consistent with previous reports of reliable repair performance using contemporary universal adhesives [44–46]. The absence of a significant bond-strength advantage for the ARS-containing adhesive and the lack of benefit from separate silanization further suggest that additional silanization may not be necessary when an effective surface pretreatment and universal adhesive are used [17, 35, 47]. The present study extends this evidence by systematically evaluating mechanical pretreatment, acid conditioning, and adhesive strategy within the same factorial framework.

Several limitations should be acknowledged. First, the in vitro design cannot fully reproduce the thermal, mechanical, enzymatic, and chemical challenges present intraorally. Second, thermocycling and long-term post-repair aging were not performed, although the original SFRC substrates underwent 3 months of hydrolytic aging before repair. Third, surface morphology, roughness, wettability, and chemical composition after treatment were not directly characterized; therefore, the proposed mechanisms should be regarded as hypotheses supported by previous literature rather than directly demonstrated mechanisms. Finally, only one SFRC material and selected adhesive systems were evaluated, limiting generalizability to other materials and formulations. Future studies should assess long-term repair durability following thermomechanical aging and evaluate a broader range of SFRC materials and repair systems.

Collectively, the present findings support the potential use of a simplified repair protocol incorporating mechanical surface pretreatment, phosphoric acid conditioning, and universal adhesive application for aged SFRC restorations. Hydrofluoric acid conditioning was associated with substantially lower repair µTBS than phosphoric acid conditioning, while neither additional silanization nor the use of an ARS-containing adhesive provided a significant bond-strength advantage. These findings may support a simplified repair workflow under the conditions evaluated.

## Conclusions

Within the limitations of this in vitro study, the repair bond strength of hydrolytically aged short fiber-reinforced resin composite was significantly influenced by the repair protocol employed. The air abrasion + phosphoric acid + universal adhesive protocol demonstrated superior repair bond strength and the most favorable failure-mode distribution. Additional silane application did not significantly improve repair performance, while ARS-containing adhesive did not demonstrate a significant bond-strength advantage under the conditions tested. In contrast, hydrofluoric acid-treated protocols demonstrated significantly lower repair bond strength and resulted predominantly in adhesive failures. The findings support the potential use of a simplified repair protocol incorporating mechanical surface pretreatment, phosphoric acid conditioning, and universal adhesive application for aged short fiber-reinforced resin composite restorations.

## Data Availability

The datasets generated and/or analyzed during the current study are available from the corresponding author upon reasonable request.
